# Studies of Resistance of PP/Natural Filler Polymer Composites to Decomposition Caused by Fungi

**DOI:** 10.3390/ma14061368

**Published:** 2021-03-11

**Authors:** Anna Włodarczyk-Fligier, Magdalena Polok-Rubiniec

**Affiliations:** Department of Engineering Materials and Biomaterials, Faculty of Mechanical Engineering, Silesian University of Technology, Konarskiego 18a, 44-100 Gliwice, Poland; magdalena.polok-rubiniec@polsl.pl

**Keywords:** polymeric materials, WPC, biodegradation, hazelnut shell flour, walnut shell flour, fungi

## Abstract

The article discusses the grain morphology of the natural filler from hazelnut and walnut shell flour. It was observed that the geometry of both meals is similar to each other and resembles uneven balls in shape. The heterogeneity and well-developed outer surface of the flour grains allow for filling the voids with the polymer matrix. The analysis of the surface of the SEM images allowed to observe the presence of natural filler flour grains in the entire volume of the produced polymer composites, uneven distribution and small agglomerates, as well as the presence of voids, distributed in the matrix and in the matrix/filler interface. As a result of the visual evaluation of the activity of microorganisms (mycelium) on the surface of the produced polymer composite materials PP/hazelnut and walnut shell flour with a different % share, different fraction, it was found that the best fungistatic effect was shown by the samples marked with the symbol hazelnut at the fraction 315–443 µm. The least fungistatic material was found to be the samples with walnut shell meal filler at the fraction 315–443 µm (F2 and F4), on which the microorganisms achieved significant growth (more than 50% of the test area). The highest value of contact angle was obtained for samples with hazelnut filler fraction 315–443 (C2 and C4), which also confirms its best fungistatic effect.

## 1. Introduction

Over the past few years, interest in polymeric WPC composite materials has increased, resulting in broader research into modifying these materials. Modern materials are produced with increasingly better properties, containing natural plant fillers. 

Until now, wood has been the main natural construction material owing to its high inherent strength, and the fact that they are easily shaped and machined by uncomplicated, inexpensive methods using versatile tools. Unfortunately, wood has the disadvantage of being flammable, as well as being susceptible to moisture and biological microorganisms. This is very important when using wood for structures located outdoors, which necessitates regular impregnation. Naturally, there are defects in wood in the form of knots, which also adversely affect the properties of products made from wood [1,2]. The United States is the largest market for the production of WPC composites using wood flour as the filler. This country produces mainly board-like profiles to replace wooden boards in outdoor applications (porches, terraces, and balustrades). In Germany, this type of material is used in the automotive industry, and in China, the applications are most varied, from production of elements of window profiles, doors, flower pots, benches to thermal insulation systems [3,4,5,6]. WPC materials can also be used in sports equipment, such as baseball bats and skis [7,8,9,10,11,12,13]. Inedible parts of fruits or plants, cereal grains, plant stalks, nutshells may be used as an alternative to wood flour used in polymer composites, which makes it possible to obtain the product features desired by the consumer, such as: Aesthetics, low product weight, high resistance to UV factors or moisture.

The use of hazelnut or walnut shell flour as a filler may reduce water absorption and improve the functional properties of the products, which would make it possible to use them, among other things, for garden elements (terrace boards, balustrades, playgrounds, roofing, etc.) [14,15,16,17,18,19,20,21,22,23].

In many countries, nutshells are crushed, and this reduces the cost of the process compared to wood, which is a significant advantage for using nut flour as a filler in WPC materials. In addition, nutshell flour has a relatively low price, about 5 cents/kg lower than the cheapest wood flour—pine flour. These features make the study of WPC composites filled with nutshell flour a response to real consumer needs [23].

However, ongoing research on these materials shows that the use of natural fillers reduces the usable life of products made from them, as they are exposed to microorganisms, mainly fungi and bacteria, which thrive in environments suitable for them. Their rapid development has a strong influence on the deterioration of product properties, and therefore, an assessment is carried out in terms of the factors affecting these properties.

The result of research on biological resistance of polymer composite materials is the assessment of the susceptibility of such materials to microbial colonization processes. At a given time, the speed and size of the area occupied by fungal mycelium is checked [24].

Fungal growth on materials is a type of biological corrosion, the summation of two chemical processes: Biodegradation and mycotoxic environmental contamination.

Biodegradation is the so-called decomposition of plastics and polymeric materials in the natural environment as a result of the activity of microorganisms [24,25,26,27]. 

Adequate access to nutrients as well as favorable conditions, i.e., adequate humidity (approx. 60–70%), temperature and weather conditions, contribute to the proper development of fungi. The type of medium on which the fungi grow depends on the type of fungi growing most commonly.

The rate, nature, and growth of microorganisms affect the performance, chemical, physical and strength properties of both plastics and polymeric composite materials with plant fillers. 

The impact of microorganisms on polymer composites involves a number of chemical and biological processes. Enzymes produced by microorganisms shorten the polymer chains in the material, reducing the molecular weight, altering a number of properties, and thus affecting other degradation processes [24,28].

The biodegradability of polymeric materials depends on their intended use. On the one hand, the aim is to develop materials with increased biodegradability, and these are all kinds of packaging, while on the other hand, materials with reduced microbial activity are being developed in order to extend their useful life, e.g., materials for construction, the automotive industry, or the decorative and utility industries.

This paper presents the results of: Morphology of the natural filler grains, microstructure of the produced polymeric composites, and a visual assessment of the surface of the produced PP polymeric composite materials/hazelnut shell flour and walnut shell flour in terms of mycelium and wetting angle.

## 2. Materials and Methods

The investigations were carried out on polymer composite materials based on the Moplen HP400R polypropylene (produced by Lyondellbasell in Poznań, Poland) matrix with the filler from the hazelnut and walnut shell flour walnut shell flour with 30% and 50% content and fractions of 0–200 µm and 315–443 µm (Table 1). The PP polypropylene used as a matrix is a homopolymer designed for injection, characterized by low viscosity and good stiffness. 

Before extrusion, the hazelnut and walnut shells used as a filler to produce polymeric composite materials were ground in a two-step process: Stage I in a Schutte Buffalo hammer mill (Instytut Nowych Syntez Chemicznych. Oddział Chemii Nieorganicznej “IChN”, Gliwicach, Poland) and Stage II in a turbine mill (Instytut Nowych Syntez Chemicznych. Oddział Chemii Nieorganicznej “IChN”, Gliwicach, Poland). After grinding, the resulting flour was passed through sieves with different mesh sizes in order to obtain different flour fractions. 

Then, the flour was dried at a temperature of about 80 °C for about 4 h and mixed with polyolefin granulate. Such blends were subjected to one homogenizing extrusion using a Goöttfert counter rotating twin-screw extruder (Sieć Badawcza Łukasiewicz–Instytut Inżynierii Materiałów Polimerowych i Barwników, Gliwice, Poland) at a ratio of L/D = 25, equipped with an extrusion head for extruding a bar with ø 3 mm diameter at the exit. 

The extrusion process conditions selected for the PP/nut shell flour composite blends: Zone I temperature: 180 °C, zone II temperature: 200 °C, zone III temperature: 210 °C, head temperature: 215–220 °C, number of rotations: 2–4 rpm.

After extruding, the granulate was injected, and the final results were test samples in the form of a standardized A1-type test piece according to PN-EN ISO 527–1 [29]. The injection process was carried out with a Battenfeld Plus 35/75 injection molding machine (Sieć Badawcza Łukasiewicz–Instytut Inżynierii Materiałów Polimerowych i Barwników, Toruń, Poland) equipped with a Unilog B2 control system with the L/D ratio of 17. 

### 2.1. Structure Study 

Images of the microstructure of the surface of natural filler powders and the surface structure of the produced polymeric composite materials were made using a SEM scanning electron microscope type ZEISS Supra 35 (The Silesian Technical University, Gliwice, Poland), at magnifications of 100× and 200× for the filler, and 100× for the produced polymeric composites. Before examination on the microscope, all flour samples and composite samples were placed on carbon tape and dusted with gold.

### 2.2. Influence of Microorganisms on the Surfaces of Polymer Composite Materials

The evaluation of the impact of microorganisms on the surfaces of manufactured polymeric composite materials was carried out in accordance with PN-EN ISO 846, 2002 [30]. The methods used were Method A of the standard “Mycelium growth test” for the determination of the resistance of plastics to fungi and Methods B and B’ “Determination of the fungistatic effect.”

Fungal reference strains were used for the study: *Aspergillus niger* (ATCC 6275), *Chaetomium globosum* (ATCC 6205), *Gliocladium virens* (ATCC 9645), oraz *Paecilomyces variotti* (ATCC 18502).

The size, shape of the test specimens, materials, test methodology and guidelines described in EN ISO 846, 2002 were applied to the tests.

In Method A, samples are exposed to a suspension of a fungal spore mixture in the presence of an incomplete medium (no carbon source). Fungi can only grow at the expense of the test material. If the samples do not contain nutrient elements, no fungal growth can occur, and the properties of the plastic will not deteriorate as a result. This method is used to assess the natural resistance of plastics to fungi where no other organic matter is present.

In Methods B and B’, samples are exposed to a suspension of a fungal spore mixture in the presence of a complete medium, i.e., with a carbon source. Even if the material does not contain any nutrients, fungi can grow on the samples and their metabolic products can affect the material. In the case of Method B’, samples are not placed on the medium until the medium is fully grown.

According to EN ISO 846, 2002, a division into 3 batches of samples was used:

- Batch 0—control samples, kept at a standardized temperature and relative humidity, (23 ± 1 °C, 50% humidity ± 5);
- Batch I—samples seeded with micro-organisms and incubated at 24 ± 1 °C;
- Batch S—non-seeded samples, stored under the same conditions as Batch I.

Visual assessment was carried out according to the scale contained in the standard (Table 2, after 4 weeks of incubation).

### 2.3. Surface Observation

Images taken with the SCAN^®^ 1200 (Interscience, France). automatic colony counter were used for visual assessment. For microscopic observations, an Olympus SZX 12 stereo microscope (Olympus, Tokyo, Japan) was used at 60× magnification of the sample image, using a camera ARTRAY Model ARTCAM 300MI (Artray, Tokyo, Japan).

### 2.4. Wettability 

The wettability of surface of tested polymer composites was determined on the base of contact angle measured by direct method. Deionized water with a surface tension of 72.8 mJ/m^2^ was used for measurements. Investigations were made in the air with a humidity of approximately 30% at room temperature using a G-10 device Krüss GmbH Company (Hamburg, Germany), equipped with PG software. Water drops were placed on surfaces of tested samples and the contact angle was then measured. For this, 10 measurements were made for each sample. 

## 3. Results and Discussion

**Figure 1:**
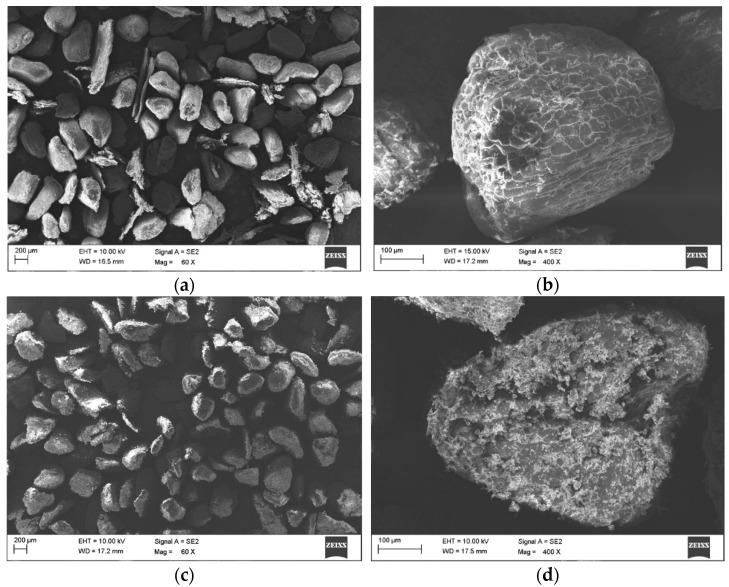
Photos of hazelnut shell meal (**a**,**b**) and walnut (**c**,**d**).

The grain morphology of the natural plant fillers was assessed from the analyzed SEM images. This analysis (Figure 1) confirmed that the hazelnut and walnut shell flour grains in the loose state obtained by milling differed in surface structure, while their geometry was similar. The particles of the ground shells resembled uneven balls (Figure 1b–d) and, moreover, their surface was rough, but when comparing the grains from the shell flour of the two types of nuts, the surface of the walnut particles appears to be more rough and uneven. The heterogeneity and well-developed outer surface and the discontinuities seen within the grains are most likely due to the structure of the nutshells. These discontinuities allow the free spaces to be filled by the PP matrix, which affects its bonding to the filler [31,32,33,34].

Hazelnut and walnut shell flour are a promising filler for polymeric composite materials. This is owing to their favorable structure. Nutshells are made up of sclerenchyma, which is dead tissue, and its function is to strengthen the plant. Mature sclerenchymatous cells have strongly thickened secondary walls and are the hardest plant tissues, characterized by very high hardness and rigidity and low water absorption. In addition, suberin is found in the nutshells. This substance is composed primarily of long-chain fatty acids and their esters with aliphatic alcohols, which exhibit strong hydrophobic properties that further protect the shell against biodegradation and moisture absorption. The hydrophobic nature of the shell surface presents considerable difficulty in breaking down this plant tissue by microbial enzymes and assimilating it. There are practically no natural polymers in them: Lignin and cellulose, which can degrade quite easily in wood under the influence of various environmental factors, e.g., UV radiation, which leads to wood graying and color change. The absence of lignin and cellulose in the nutshells has another very important advantage from the point of view of thermoplastics processing. It improves the thermal resistance of the filler in the matrix. Possessing such features allows the creation of new WPCs (wood-polymer composites) composite materials devoid of the basic disadvantage of this type of material, which is the change of properties and reduction in durability under the influence of moisture and microorganisms [35,36].

**Figure 2:**
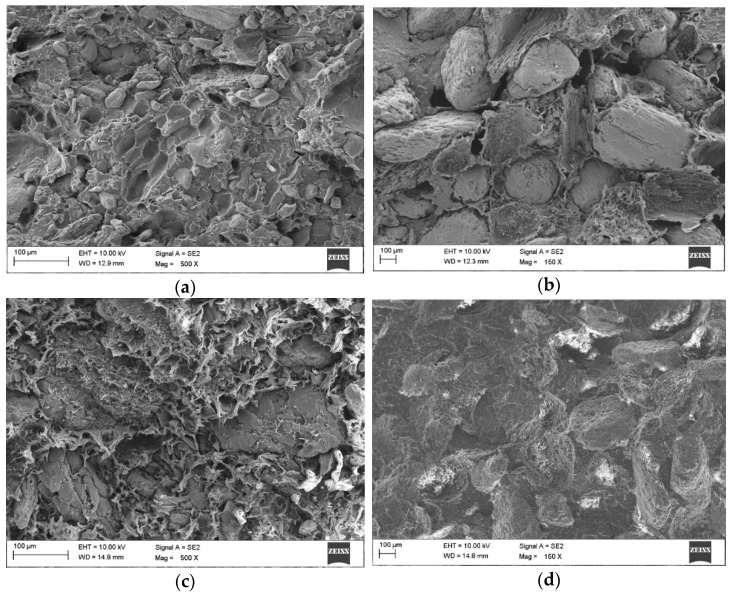
Photo of the produced PP/shell flour polymer composite material: (**a**) A4 and (**b**) C4 from hazelnuts and (**c**) D4 and (**d**) F4 from walnuts.

The surface analysis of SEM images allowed to observe the presence of natural filler flour grains in the whole volume of the produced polymer composites, as well as their uneven distribution and small agglomerates (Figure 2). The uneven distribution of fillers in the PP matrix is characteristic of particle-filled polymer composites [10,34]. Proper wettability of the filler surface resulting from the use of a relatively low molecular weight polymer increases the adhesion between the matrix and the filler [16,34]. The PP polymer matrix not only covers the surfaces of the plant filler flour but also appears to penetrate deep into the porous grain structure. The presence of voids, free spaces distributed in the PP matrix, as well as in the matrix/filler interface, was also observed. 

The higher porosity of the material was observed in composites filled with hazelnut shell flour, regardless of the % amount of the filler, which is most likely due to the smoother surface structure of the hazelnut shell, which may result in limited adhesion between the matrix and the natural filler. A few pores of larger size that can be observed in the material may be the result of impeded degassing of the highly viscous components, which is influenced by the shape and surface of the flour grains [16,34,37].

The susceptibility of manufactured polymeric composite materials to microorganisms depends, among other things, on the type of polymer, the natural filler, the % share of the filler in the matrix, and the particle size of the filler. Among other things, this is related to the properties of the PP polymer, i.e., its molecular weight, chemical structure, and physicochemical properties [24,38,39]. Synthetic polymers including PP show little susceptibility to microorganisms as a result of the hydrophobic chemical structure of the surface [24,40].

The results of the visual analysis of the individual fungal treated samples are included in Table 3, Table 4 and Table 5.

**Figure 3:**
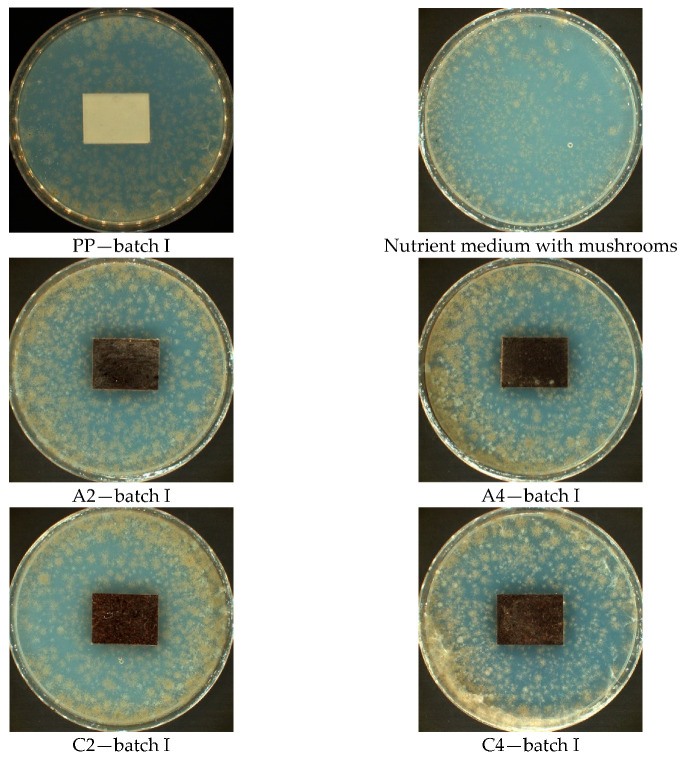

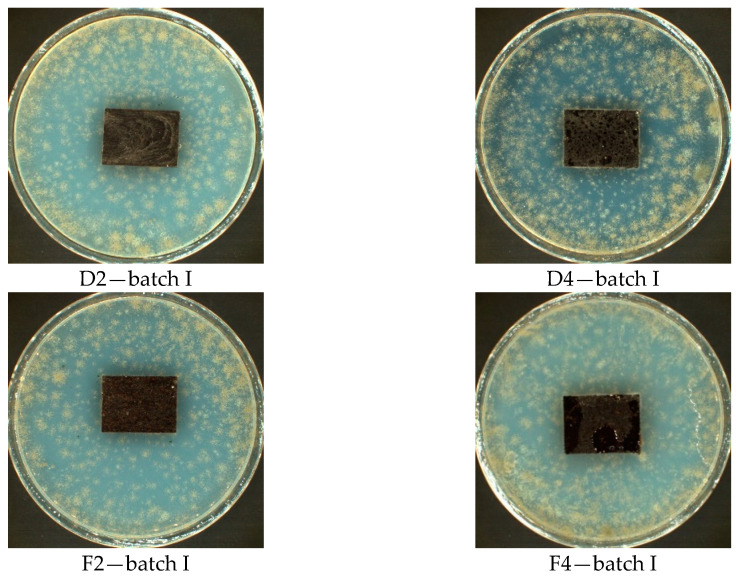
Method A—results of visual assessment of samples; photos of batch samples: I.

In the case of walnut filler (D2, D4, F2, and F4), in both fractions (0–200 µm and 315–443 µm) with different % (30% and 50%) amount in Method A, where samples are exposed to a suspension of a mixture of fungal spores in the presence of an incomplete nutrient medium (without a carbon source), significant mycelium growth was observed, covering more than 50% of the test area samples (Figure 3). In this method, mushrooms can only grow at the expense of tested material. 

**Figure 4:**
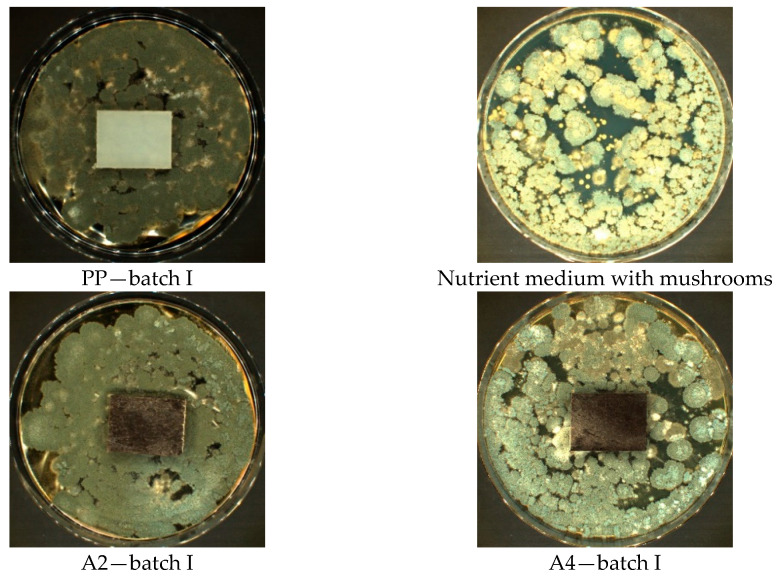

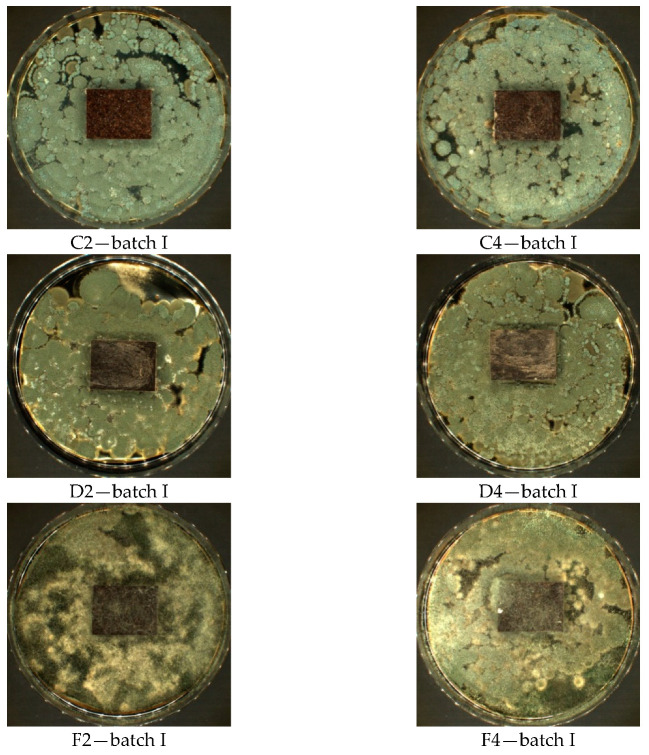
Method B—results of visual assessment of samples; photos of batch samples: I.

**Figure 5:**
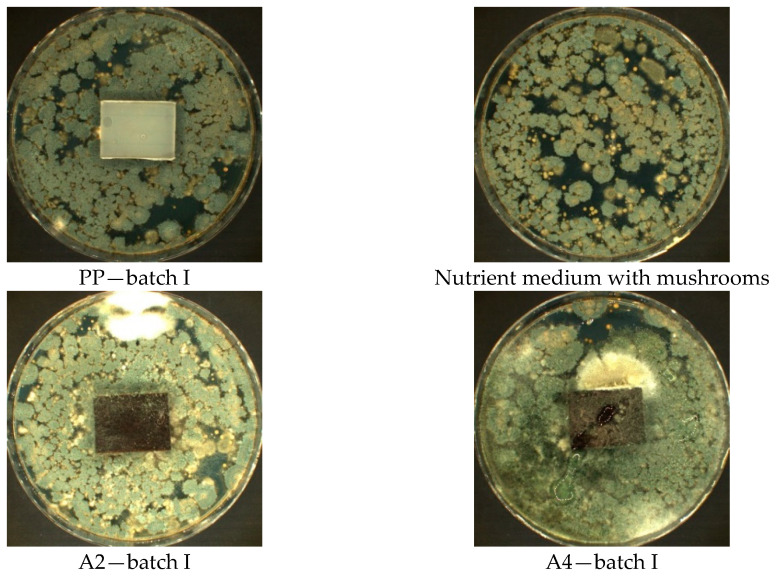

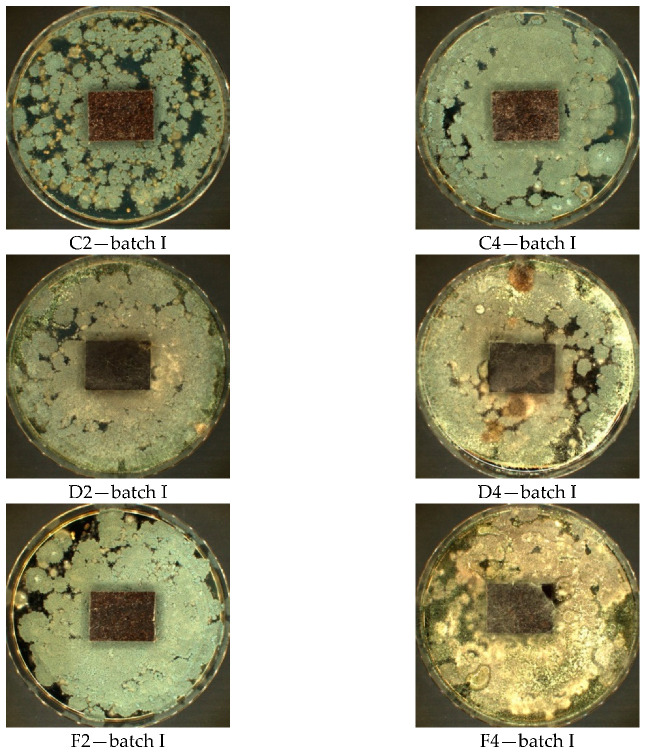
Method B’—results of visual assessment of samples; photos of batch samples: I.

On the other hand, in Method B and B’, where samples are exposed to a suspension of mixture of fungal spores in the presence of a complete nutrient solution, i.e., with a carbon source, in the case of a larger fraction in both cases of natural walnut filler (315–443 µm), intensive fungal growth was observed, covering the entire test area (Figure 4 and Figure 5). 

The best fungistatic effect demonstrated samples marked symbol C2 and C4 (hazelnut meal filler) in all Methods A, B, and B’. With the naked eye, mycelium growth was observed up to 25% of the tested area, with both 30% and 50% filler content and the size of the meal fraction 315–443 µm. The other tested materials marked A2, A4, D2, D4, F2, F4 (Method A) did not show resistance to fungi. The fungal colony growth was observed to cover more than 50% of the tested area. The intensive growth of the mycelium covering the entire surface of the tested samples took place on the composites designated F2 and F4 (Method B and B’).

The least fungistatic material of all tested samples were samples with 30% and 50% walnut shell meal filler and 315–443 µm fraction (F2 and F4), on which the microorganisms achieved significant growth covering the entire surface area. This is probably due to the structure of shell being rougher and uneven than that of hazelnut shells, where this fungus resistance is the best in larger fraction. 

**Figure 6:**
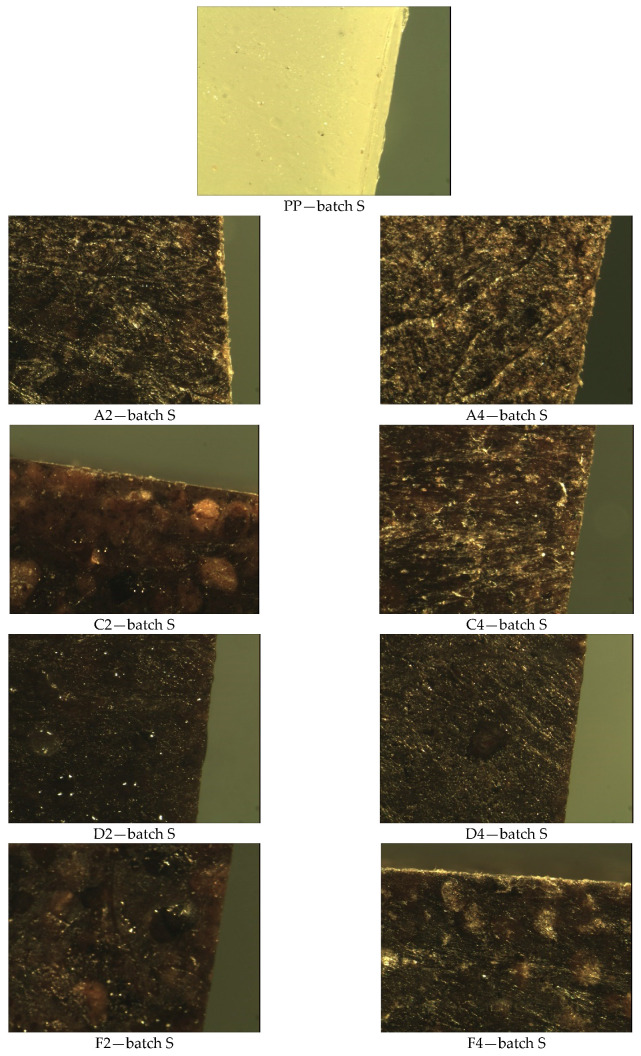
Results of microscopic evaluation of batch samples: S.

Figure 6 shows the surfaces of the tested samples not inoculated with the mixture of fungal embryos in which no mycelial colonies were observed.

**Figure 7:**
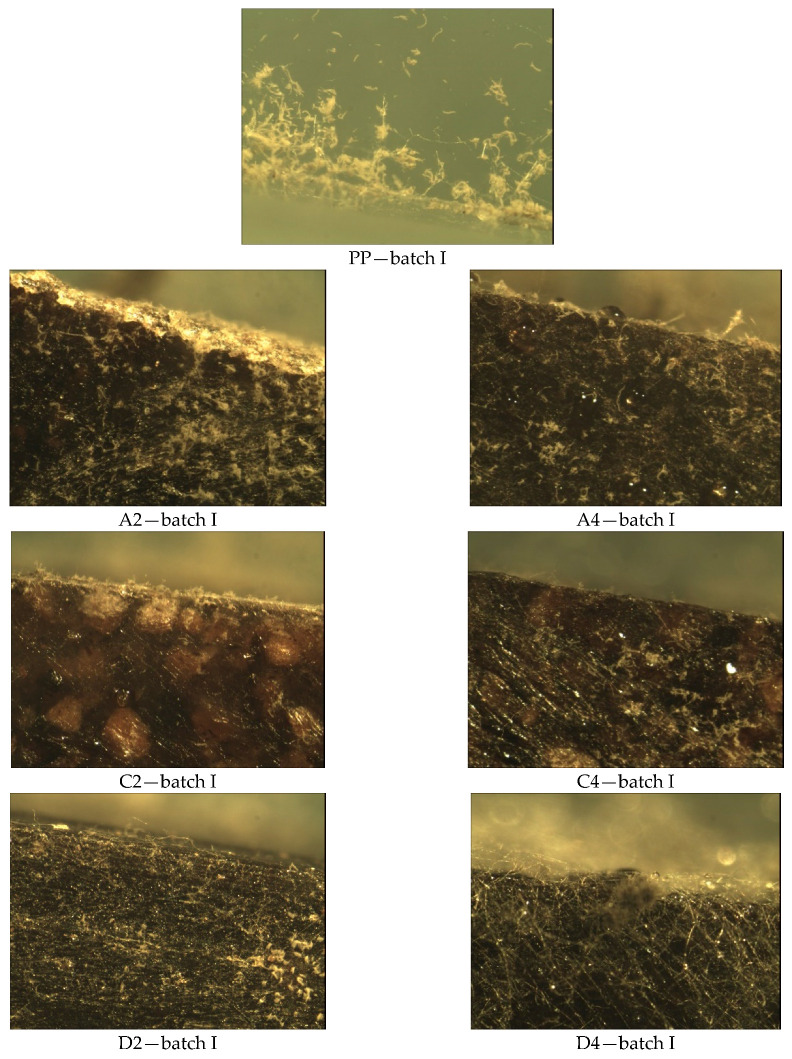

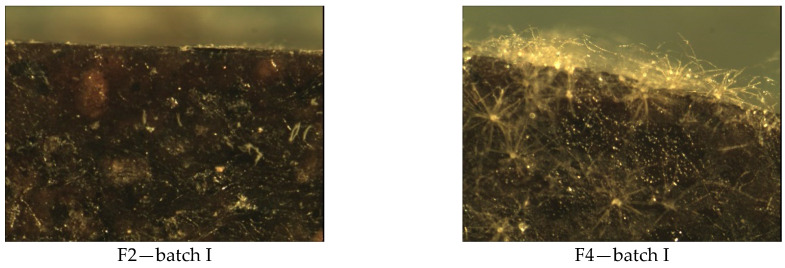
Methods A—results of microscopic evaluation of batch samples: I.

In Method A (Figure 7), where the samples were exposed to a suspension of the mixture of fungal spores in the presence of an incomplete medium, growth of fungal colonies was observed on all tested surfaces. They are visible by the naked eye and cover up to 25% in the case of samples with the symbols PP, C2, and C4. On the surface of the A4 sample with the content of 50% hazelnut meal filler and 0–200 µm fraction, water droplets and small amounts of fungal hyphae are visible. The remaining surfaces of the test samples, both with walnut and hazelnut meal filler (A2, D2, D4, F2, and F4) are largely covered (over 50% of the surface).

**Figure 8:**
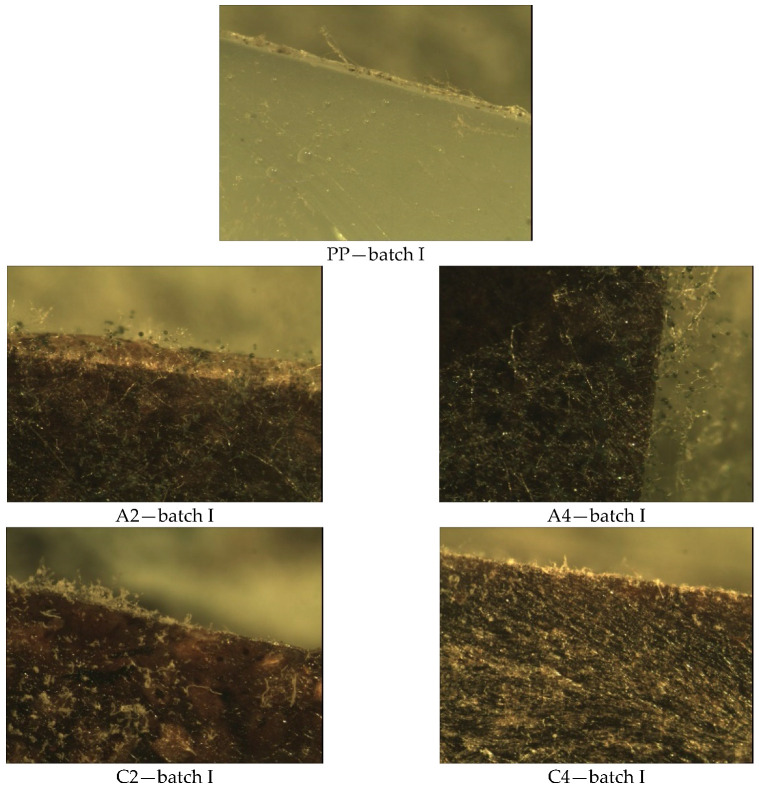

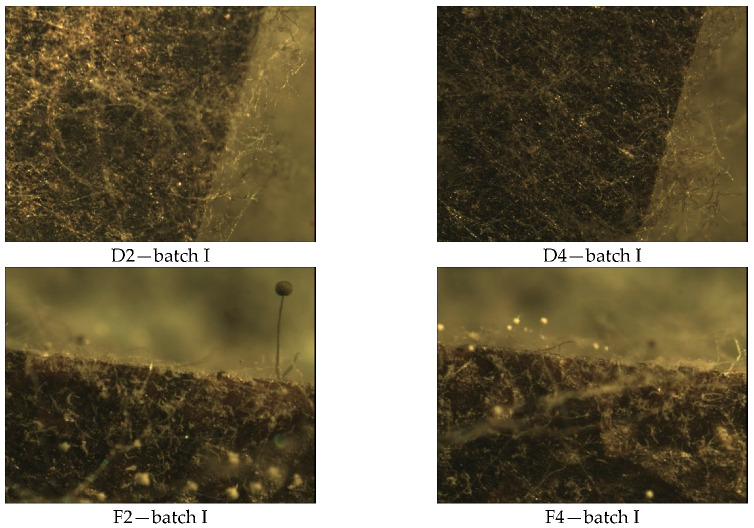
Methods B—results of microscopic evaluation of batch samples: I.

In Method B (Figure 8), where the test surfaces were exposed to a suspension of the mixture of fungal spores in the presence of a complete nutrient medium with a carbon source, a significant growth of filamentous hyphae was observed on almost the entire surface on samples A2, A4, D2, D4, F2, and F4. There are also numerous long, straight fungal hyphae elongated upwards with spherical embryos on the surface. In the case of the sample area C2 and C4, the coverage with fungal colonies is the lowest and amounts to 25%.

**Figure 9:**
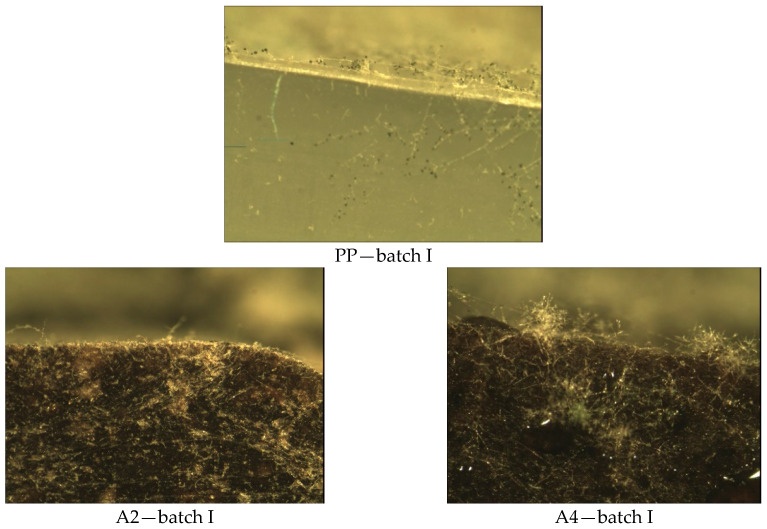

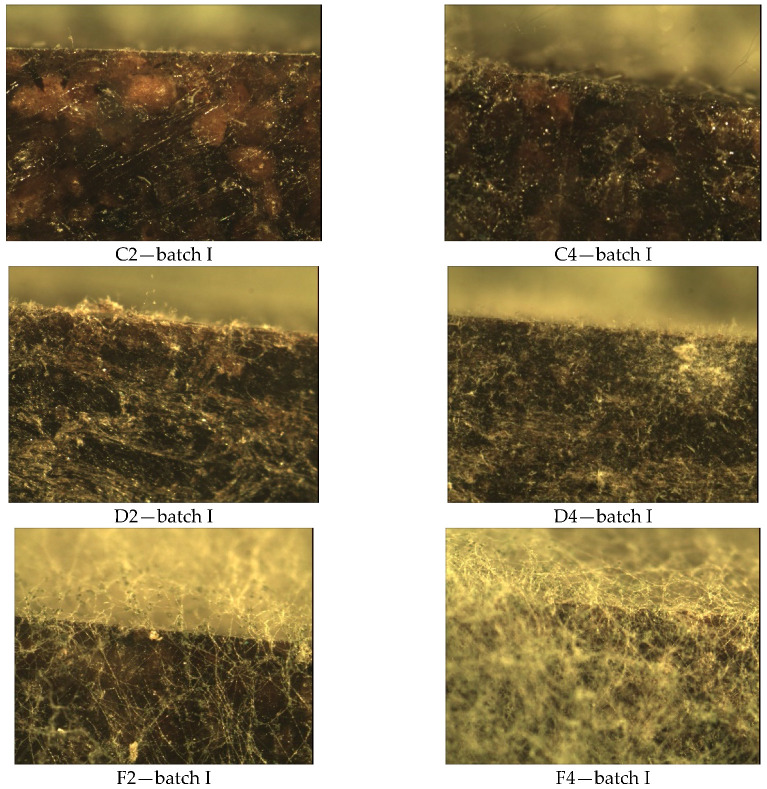
Methods B’—results of microscopic evaluation of batch samples: I.

In Method B’ (Figure 9), samples are not placed on the medium until it is completely grown. On the surface of the tested samples A4, F2, and F4 (50% hazelnut flour content with 0–200 µm fraction and 30% and 50% walnut flour content with 315–443 µm fraction), there is an intense growth of flat, thread-like hyphae colonies. In the case of samples C2 and C4, this coverage is the smallest and covers only 25% of the tested area.

In the case of the walnut filler, significant mycelial growth was observed at both fractions and % share in Method A, covering more than 50% of the surface of the examined sample. In contrast, in Methods B and B’, intensive fungal growth was observed for the larger fraction (315–443 µm), covering the entire examined surface.

Visual observation of the mycelium growth process on the surface of the tested materials showed that both the environment in which the tests were carried out, the type of filler (hazelnut and walnut flour), the grain fraction, and the percentage of flour in the polymeric composite materials produced had an effect on the result. Visible fungal culture colonies can be observed on samples in Method A, B, and B’ (Figure 3, Figure 4, Figure 5, Figure 6, Figure 7, Figure 8, and Figure 9). 

The measure of wettability of tested surface is contact angle, also known as wetting angle. It is angle between tested surface and the tangent to the surface of distilled water drop wetting the surface. The value of contact angle lower than 90° determines the degree of wetting of tested surface. A contact angle of 0° theoretically defines the ideally spreading liquid over surface and complete wetting. A contact angle of 180° corresponds to the complete non-wettability of the surface. 

The wettability result is influenced by factors such as: Surface heterogeneity under chemical or physical influence, surface roughness, contamination on the surface, and type and size of the measuring drop. Table 6 below shows values of contact angle of tested surfaces. 

The highest values of contact angle were shown by polymer composites marked symbol A2, C2, and C4 (100.02°–105.36°), which proves their worse wettability in comparison with wettability of other composite surfaces (PP, A4, D2, D4, F2, and F4). In all cases, the contact angle is close to 100°, which may prove stable, homogeneous energy properties of tested surfaces in the area of contact with water drop. 

Samples marked F2 and F4 showed the lowest contact angle and the differences between samples with different percentages were the smallest in comparison with other samples, which proves the best wettability. Samples marked A2, A4, C2, and C4 showed the lowest values of standard deviation of contact angle (1.29°–2.64°), which may result from the high dimensional stability of droplet or the lack of a tendency for water droplets to spread on tested surfaces. 

The differences between the wettability of tested samples with the same fillers, but with a different content in the PP matrix (97.55°) and with a different fraction, is small. 

The wettability of samples marked F2 and F4 (97.85°–97.14°) is the closest to the contact angle of PP matrix surface itself. The difference in contact angle of tested samples depends on the type of natural filler, the amount of % filler in matrix, as well as its fraction.

## 4. Conclusions

The activity of microorganisms, first of all fungi, significantly affects the specific features and utility of tested polymer composites exposed to contact with microorganisms. Biological corrosion caused by the growth of fungi takes place when favorable conditions are met.

Tested polymer composites contain nutrients in the form of natural fillers, such as walnut or hazelnut shell flour. It was possible to assume that they are susceptible to the action of microorganisms, which was confirmed by the research results obtained and described in the paper.

Despite the use of PP matrix, which is characterized by a relatively low molecular weight, which increases adhesion with the filler, and PP is a type of polyolefin with high resistance to biodegradation, it did not form a sufficiently tight cover around the filler particles to protect the material from mycelium.

The occurrence of pores observed in composite matrix, regardless of the % amount of natural filler, probably results from difficult degassing of sticky matrix and the filler components, which also affects the resistance to microorganisms.

There are many methods available for testing fungus degradation of polymer composites, but currently there is no laboratory standard for testing.

As a result of visual evaluation of the effect of microorganisms—fungi, on the surface of manufactured PP polymeric/nutshell flour composite materials with different % share of different fractions, it was found that the best fungistatic effect was demonstrated by the samples labeled C2 and C4 in all Methods A, B, and B’. The other tested materials, A2, A4, D2, D4, F2, and F4, did not demonstrate any fungal resistance. Intensive microbial growth covering the entire surface was noted (for Methods B and B’). The least fungistatic material proved to be samples with walnut shell flour filler at 30% and 50% share and fractions of 315–443 µm (F2 and F4), on which microorganisms reached (after 4 weeks of testing) a significant growth, covering more than 50% of the examined surface. This could probably be due to the rougher and more uneven shell surface compared to hazelnut shells, where this fungal resistance is best with a larger fraction.

Both in Methods A, B, and B’, the growth of fungal colonies to a different degree was observed on the examined surfaces. In Methods A and B, numerous hyphae elongated upwards ended with embryos were observed on the surface of samples A, D, and F. On the other hand, the surfaces of samples C2 and C4 are the least covered in all the Methods.

Interaction between polymer matrix and natural filler is of great importance. Natural fillers used in the form of hazelnut and walnut shell flour are characterized by a highly lignified cell structure, which is the main component of walnut shells. Almost 90% of cell’s volume is attributed to the walls, thanks to which shell has a better resistance to degradation than wood.

The susceptibility of manufactured polymer composite materials to fungi depends, inter alia, on the type of natural filler, the percentage of filler in matrix, and the size of its particles. The fact that surface of natural fillers is not covered by PP matrix promotes growth and spread of colonies of microorganisms, including fungi. The materials can be secured with additional agent to protect them against damaging effects of fungi, but this would increase their price and reduce the recycling process carried out after the period of use.

The hydrophilicity of tested samples determines the wettability of their surface, which will result in a good, enabling migration of fungal colonies on tested surface. The highest value of the contact angle was obtained for samples marked C2, C4, which also confirms its best fungistatic effect, which may also result from the developed surface of filler meal. The adhesion of mycelium to tested surface is conditioned by the existence of electrostatic, hydrophobic interactions between fungi and surfaces of composite materials. It was found that there is a direct influence of composition of composite material, the % amount of filler in matrix, and filler fraction on the value of water contact angle.

## Figures and Tables

**Table 1:** Marking of the investigated materials.

Flour from Walnut Shells, Fraction µm	The Content of Flour in the Composite, %					
	Hazelnut Shell			Walnut Shell		
	0	30	50	0	30	50
0–200	PP	A2	A4	PP	D2	D4
315–443	PP	C2	C4	PP	F2	F4

**Table 2:** Evaluation of microbial growth according to EN ISO 846 (2002).

Growth Intensity	Rating
0	No visible growth under the microscope.
1	Growth invisible to the unaided eye but clearly visible under the microscope.
2	Growth discernible to the unaided eye, covering up to 25% of the examined area.
3	Growth discernible to the unaided eye, covering up to 50% of the examined area.
4	Substantial growth, covering more than 50% of the examined area.
5	Intense growth, covering the entire examined area.

**Table 3:** Fungal growth on test samples (Method A)—result of visual assessment of samples based on Table 2.

Tested Samples									
Batch	PP	A2	A4	C2	C4	D2	D4	F2	F4
0	0	0	0	0	0	0	0	0	0
I	2	4	4	2	2	4	4	4	4
S	0	0	0	0	0	0	0	0	0

**Table 4:** Fungal growth on test samples (Method B)—result of visual assessment of samples based on Table 2.

Tested Samples									
Batch	PP	A2	A4	C2	C4	D2	D4	F2	F4
0	0	0	0	0	0	0	0	0	0
I	1	4	4	2	2	4	4	5	5
S	0	0	0	0	0	0	0	0	0

**Table 5:** Fungal growth on test samples (Method B’)—result of visual assessment of samples based on Table 2.

Tested Samples									
Batch	PP	A2	A4	C2	C4	D2	D4	F2	F4
0	0	0	0	0	0	0	0	0	0
I	1	4	5	2	2	4	4	5	5
S	0	0	0	0	0	0	0	0	0

**Table 6:** Results of the wetting angle.

Designation of the Sample	PP	A2	A4	C2	C4	D2	D4	F2	F4
Wetting angle (°)	97.55 ±6.95	104.81 ±1.29	98.22 ±2.64	100.02 ±1.47	105.36 ±1.85	97.33 ±8.27	101.35 ±5.88	97.85 ±2.37	97.14 ±2.40

## Data Availability

The data presented in this study are available in the article.
